# A Comparative Study of Radiography and Computed Tomography in Detecting Periapical Infections in Alpacas (*Vicugna pacos*)

**DOI:** 10.3390/ani15213138

**Published:** 2025-10-29

**Authors:** Linda Rutigliano, Els Raes, Kirsten Proost, Lieven Vlaminck, Katrien Vanderperren

**Affiliations:** 1 Department of Morphology, Imaging, Orthopedics, Rehabilitation and Nutrition, Faculty of Veterinary Medicine, Ghent University, 9820 Merelbeke, Belgium; linda.rutigliano@ugent.be (L.R.); els.raes@ugent.be (E.R.); 2 Wooly Veterinary Dentist, 9810 Nazareth-De Pinte, Belgium; 3 Department of Surgery and Anesthesiology of Domestic Animals, Faculty of Veterinary Medicine, Ghent University, 9820 Merelbeke, Belgium

**Keywords:** radiography, computed tomography, apical infection, alpaca

## Abstract

Alpacas are frequently affected by dental pathologies, with periapical infection of the cheek teeth being a significant concern. While radiography is commonly used as the primary diagnostic imaging modality, it has known limitations, particularly due to anatomical superimposition. Computed tomography (CT), with its superior spatial resolution, allows for more detailed evaluation and can detect early or subtle changes that may not be visible on radiographs. To date, direct empirical comparisons between radiography and CT in diagnosing apical infections in alpacas have not been documented. In this study, 158 teeth from 19 alpacas were assessed using both radiography and CT, evaluating 16 diagnostic parameters. Inter-modality agreement demonstrated variable consistency across assessed features: No agreement was observed for certain findings such as pulpar changes and the presence of cementomas. Agreement was weak for features including periodontal ligament space widening, periapical halo, lamina dura visibility, root lysis and fragmentation, sequestration, cortical destruction, clinical crown fractures, and the overall impression of periapical infection. Moderate agreement was found for radicular changes, periapical sclerosis, periosteal reaction, mandibular expansion, and alveolar bone alterations. Overall, CT identified more dental abnormalities than radiography, highlighting its value as a complementary diagnostic tool for detecting periapical infections in alpaca cheek teeth.

## Simple Summary

Alpacas are gaining popularity in Europe, where they are kept as pets or wool-producing animals. Their increase in number brought a new diagnostic challenge for veterinarians. Specifically, these animals are commonly affected by dental infections. The initial suspicion of dental infection is normally raised upon oral examination; however, diagnostic imaging is needed to further evaluate the portion of the dental elements that cannot be assessed upon oral examination. The imaging modalities that are commonly used for this purpose are radiography and computed tomography (CT). This study aims to compare the ability of these two modalities to detect dental infections. Overall, CT identified more dental abnormalities than radiography, highlighting its value as a complementary diagnostic tool for detecting periapical infections in alpaca cheek teeth.

## 1. Introduction

The global rise in alpaca popularity is largely due to their impressive adaptability, allowing them to flourish across a wide range of climates while requiring relatively low maintenance owing to simple management and breeding practices [1]. However, this growth in alpaca populations has introduced new challenges in veterinary diagnostics. Notably, dental disorders have become a prevalent issue in alpaca healthcare worldwide [2,3,4]. Among these, periapical infections—commonly referred to as tooth root abscesses—stand out as one of the most frequently diagnosed conditions [3,5,6]. These infections tend to involve the premolars and molars, likely due to their critical function in mastication, which subjects them to repeated mechanical stress and potential trauma [6].

In clinical settings, radiography remains the primary diagnostic imaging modality employed for the initial assessment of dental and craniofacial conditions in veterinary practice. This technique is widely accessible, cost-effective, and provides rapid visualization of hard tissues, making it a useful tool for routine screening and follow-up examinations. Recent research has provided detailed guidance on optimal radiographic techniques for evaluating dental structures in alpacas tailored to the species’ unique craniofacial anatomy [7]. These guidelines have improved diagnostic accuracy and consistency in clinical evaluations. Nevertheless, the detection of dental disease is not always straightforward with radiography alone. The complex overlapping of dental arcades, the oblique orientation of the cheek teeth, and the presence of dense surrounding bone can obscure lesions or mimic normal anatomical variations. As a result, the presence—or absence—of radiographic abnormalities often necessitates further investigation using more advanced imaging modalities, such as computed tomography (CT), which has been extensively described and validated in other species [7,8,9]. CT imaging provides several advantages, including enhanced contrast resolution, the ability to generate cross-sectional views free of superimposition, and the option for multiplanar and three-dimensional reconstructions. High-resolution CT, characterized by very thin slice thicknesses (≤1 mm), improves spatial resolution and minimizes partial volume artifacts, thereby enhancing the identification of subtle or early-stage lesions [10].

Early indicators of apical infection, as described in other species and confirmed to be similar in alpacas based on the author’s experience, typically include widening of the periodontal ligament space and thinning of the lamina dura in cheek teeth [11,12]. As the infection progresses, more radiographic changes may become apparent, such as lytic bone lesions presenting as periapical radiolucent halos or a rounded, clubbed appearance of the tooth roots [11,12,13]. Increased bone density or sclerosis in the adjacent alveolar bone is also frequently observed in later stages of the disease [11,12].

Additional radiographic indicators of apical infection include abnormal apical cementum deposition, which appears as diffuse mineralized areas surrounding the reserve crown [11,12]. In some cases, cementoma formation has been described, typically appearing as well-defined, rounded radiopaque lesions adjacent to the diseased tooth roots [13,14].

Although recent studies have improved the understanding of the normal radiographic and CT anatomy of alpaca cheek teeth [15,16,17], direct empirical comparisons between these two imaging modalities in diagnosing apical infections and sinusitis remain scarce. This study aimed to compare the detection of dental abnormalities using both radiography and CT, highlighting each method’s strengths and limitations in diagnosing such conditions. The working hypothesis was that while radiography could reveal moderate to severe dental pathology, CT would offer a more detailed evaluation and potentially reveal early or subtle changes not visible radiographically.

## 2. Materials and Methods

### 2.1. Study Population

A total of 158 cheek teeth, originating from 76 dental arcades from 19 Huacaya alpacas, were evaluated using radiographic and CT testing. The study population comprised 14 alpacas referred to Ghent University Hospital with suspected advanced dental pathology that were enrolled retrospectively. The remaining five alpacas were enrolled prospectively as cadaveric heads through the pathology department. Among these, two animals had been euthanized following a diagnosis of advanced dental disease, while the remaining three had been euthanized for reasons unrelated to dental issues. Importantly, no animals were euthanized specifically for this study. The use of live animals in this study was not subject to prior ethical approval by law (EU directive 2010/63/EU), as the diagnostic imaging data were obtained “below threshold”, i.e., as part of the diagnostic examination of the patient. For cadaveric specimens, ethical approval was waived by the ethical committee, based on European legislation (EU directive 2010/63/EU), as the images were obtained postmortem.

The study population (Table 1) consisted of 9 females, 2 males, and 8 castrated male animals. Age of the included animals ranged from 1 year to 11 years, with a mean of 5 years and 8 months ± 3 years and 2 months.

### 2.2. Oral Examination

In the 14 dental patients, examinations were performed under deep sedation using a combination of ketamine (5 mg/kg IM, Ketamidor^®^, Richter Pharma, Wels, Austria) and medetomidine (30 µg/kg IM, Domitor^®^, Orion Corporation, Orion Pharma, Espoo, Finland) or dexmedetomidine (15 µg/kg IM, Dexdomitor^®^, Orion Corporation, Orion Pharme, Espoo, Finland). Prior to any evaluation, the oral cavity was flushed thoroughly to evacuate any food particles that might have prevented a reliable evaluation of the dental arcade. A miniature pony speculum was applied (CAPPS^®^, Cortland, NE, USA), combined with a dental speculum light. Teeth and surrounding soft tissues were evaluated using a non-portable oroscope (Karl Storz, Tuttlingen, Germany). After image (CT and radiography) acquisition, the included cadaver heads were disarticulated in the temporomandibular joint, whereafter photographs of all teeth and specific abnormalities were taken (simulating an oral examination). Individual teeth were identified using the modified Triadan system [18].

### 2.3. Diagnostic Imaging

Six radiographic projections of each animal were acquired (55 kV and 6.3 mAs, Optimus, Phillips, Eindhoven, The Netherlands) under the same sedation (i.e., ketamine 5 mg/kg and medetomidine 30 µg/kg) to further evaluate the cheek teeth. Dorsoventral projections were performed with the animals in sternal recumbency [19]. Additionally, one lateral projection, two latero30°dorsal-lateroventral oblique projections (R30°Do-LVeO and L30°Do-RVeO) of the maxillary arcades, and two latero45°ventral-laterodorsal oblique projections of the mandibular arcades were obtained for each animal in left and right lateral recumbency with the dental arcade of interest closest to the plate. The built-in goniometer of the radiography source was used as a reference for the determination of specific angles. A 20 cc syringe (Terumo, Leuven, Belgium) was used as a mouth gag for all except the dorsoventral projections.

The animals, positioned in sternal recumbency, were subjected to a 4-slice CT scanner (Lightspeed Qx/i, General Electric Medical Systems, Milwaukee, WI, USA) with the following acquisition parameters: kVp 150, mA 250, FOV 400 mm, pitch 0.6, and slice thickness 0.5 mm. An edge-enhancing filter (window level 1000 and width 3500 with a 512 × 512 matrix) for better evaluation of the osseous details was applied to the raw CT data to optimize their use.

All images were assessed by two ECVDI-boarded veterinary radiologists (ER and KVDP). First, the radiologists independently reviewed the images. Then, a consensus was reached among them for each parameter on every dental element.

The following parameters were considered for both imaging modalities: widening of the periodontal ligament space (>1 mm), presence of sequesters, pulpar changes, periosteal reaction, periapical sclerosis, periapical halo, mandibular expansion, radicular lysis or fragmentation, detectable lamina dura, clinical crown fracture, cortical destruction, radicular changes (other than clubbing), changes in the alveolar bone (namely increased thickness), cementomas/hypercementosis, apical root clubbing, and finally overall suspicion of periapical infection.

In total, 158 teeth were selected as potentially diseased either upon oral examination or upon radiographic screening and were subsequently assessed on CT.

### 2.4. Statistical Analysis

**Figure 1:**
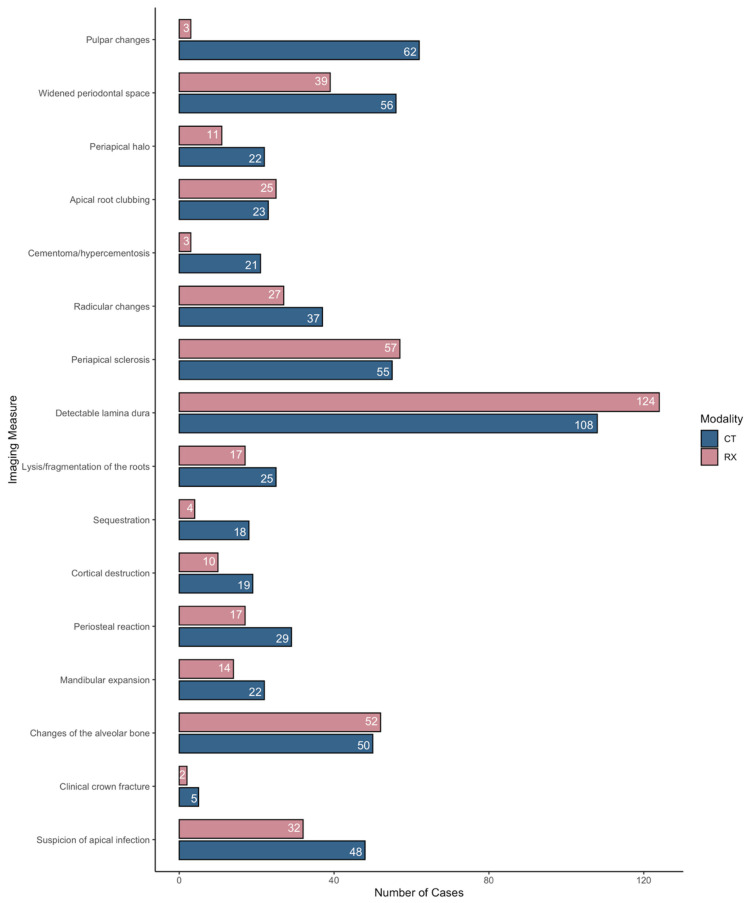
Bar plot depicting the number of abnormalities identified by each imaging modality for each of the 16 considered parameters. CT = computed tomography, RX = radiograph.

Each of the 16 parameters was scored either as present or absent using a binary code for each of the 158 teeth for each modality. All analyses were completed using R version 4.3.2. The unweighted Cohen’s kappa statistic was estimated to assess the level of agreement between the two diagnostic modalities (CT and radiology): <−0.10 = disagreement; −0.10–0.19 = no agreement; 0.20–0.59 = weak agreement; 0.60–0.79 = moderate agreement; 0.80–1 = strong agreement (Table 2 and Figure 1) [20]. Statistics were converted into percentages for easier interpretation.

## 3. Results

Results for agreement between imaging modalities for the 16 considered parameters are illustrated in Table 2 and Figure 1.

When comparing CT and radiography, no agreement was found concerning pulpar changes and the presence of cementomas/hypercementosis. Weak agreement among modalities was present for widening of the periodontal ligament space, presence of periapical halos, detectable lamina dura, lysis and/or fragmentation of the roots, sequestration, cortical destruction, clinical crown fractures, and suspicion of apical infections. Apical root clubbing, radicular changes, periapical sclerosis, periosteal reaction, mandibular expansion, and changes in the alveolar bone displayed moderate agreement between CT and radiography.

CT identified more pathological teeth compared to radiography for the following parameters: widening of the periodontal ligament space, sequester formation, pulpar changes, periosteal reaction, periapical halo, mandibular expansion, lysis or fragmentation of the roots, clinical crown fractures, cortical destruction, radicular changes, cementoma/hypercementosis, and overall suspicion of apical infection.

Radiography identified more pathological dental elements compared to CT for the following parameters: periapical sclerosis, detectable lamina dura, changes in the alveolar bone, and apical root clubbing.

## 4. Discussion

Radiography is commonly employed to diagnose cheek tooth pathology. However, due to the complex three-dimensional anatomy of the equine head, interpreting radiographs in this region can be challenging, particularly in cases of apical infection [21].

**Figure 2:**
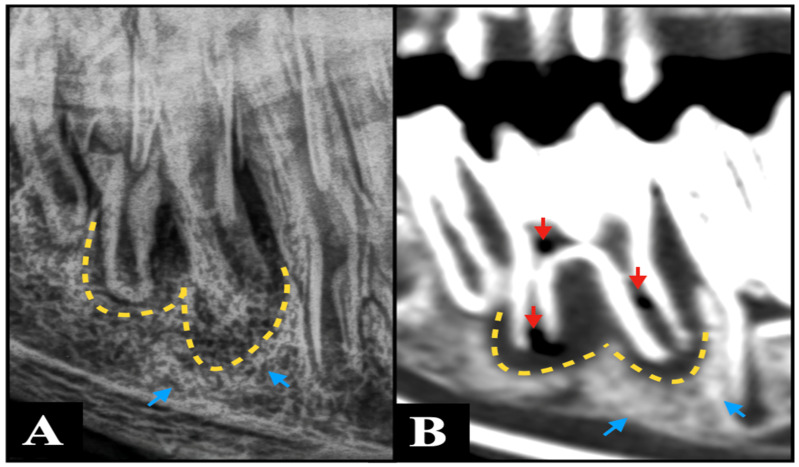
An R45°V-LDO oblique radiograph (**A**) and a bone window sagittal plane computed tomography multiplanar reconstruction image (**B**) demonstrate concordant findings consistent with periapical infection of Triadan 309, characterized by marked widening of the periodontal ligament space (yellow dotted arrow) and sclerosis of the neighboring alveolar bone (blue arrows). However, CT additionally revealed multiple gas foci within the pulp system of this tooth (red arrows)—a finding that was not detectable on the radiographic projection.

**Figure 3:**
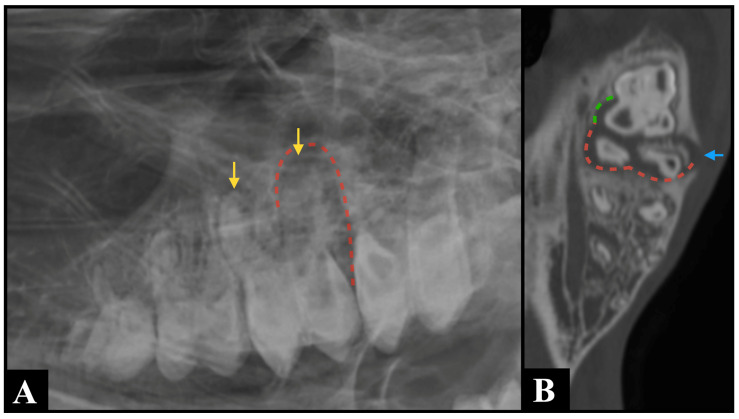
An R30° D-LVO radiographic projection (**A**) identified shortening and blunting of the root of Triadan 210 (yellow arrows), along with widening of the periodontal ligament space (red dotted line) surrounding its distal roots, which are findings consistent with a suspected periapical infection. Bone window computed tomography multiplanar reconstructed image ((**B**), dorsal plane) confirmed the suspected findings (red dotted line) and provided additional information, demonstrating involvement of Triadan 211, where the mesiopalatal root exhibited widening of the periodontal ligament space (green dotted line) and an associated complete interruption of the alveolar bone (blue arrow).

The lack of superimposition of multiple anatomical structures provided by CT is most likely the reason why widening of the periodontal ligament space, clinical crown fractures, radicular and pulpar changes, and the presence of cementomas and hypercementosis were detected more frequently by CT than by radiography in this study. Specifically, the marked over-representation of pulpar changes detected by CT compared to radiography (Figure 2) is similar to what was previously found in horses [22]. Pulpar changes included in this study were mostly represented by pulp stones, whose size makes them undetectable on radiographs. Furthermore, the possibility of performing multiplanar reconstruction is most likely responsible for the easier detection of sequestration, periosteal reaction, mandibular expansion, and cortical destruction on CT images compared to radiography. These multiplanar views allow for more precise localization and characterization of pathology, enhancing diagnostic confidence (Figure 3).

On the contrary, periapical sclerosis and other changes in the alveolar bone were more often identified by radiography than by CT. This discrepancy may reflect the influence of superimposition on radiography, which can enhance the apparent density of some structures and, in some cases, make subtle sclerosis more conspicuous than on CT.

Additionally, assessing bone involvement can be particularly challenging with radiography, as significant bone lysis (mineral content must decrease by 30 to 60% before it becomes visible on a radiograph, [23]) must occur before changes become apparent [24]. In contrast, CT and cone beam CT (CBCT) are believed to detect bone lysis changes earlier [25].

The identification of 16 additional teeth as potentially affected by periapical infection on CT compared to radiography highlights the value of CT as a diagnostic tool in dental assessments. Additionally, the possibility of performing CT under the same sedation protocol used for radiographic examination allows both procedures to be conducted sequentially during a single anesthetic event, with only minor adjustments if required. Furthermore, no additional anesthetic preparation is needed when acquiring CT images of this region compared with radiography. However, the use of CT is not without drawbacks, including the high initial and maintenance costs of the equipment and facility, the need for multiple personnel to perform the scan, and the significant expertise and time required to interpret the results [19].

In this study, CT identified a higher number of abnormalities compared with radiography, consistent with previous findings in human studies [8,9,26,27,28], where both conventional CT and cone beam CT (CBCT) have been shown to outperform intraoral radiography (IOR) in detecting periodontal and endodontic diseases. The superior sensitivity of CT is primarily due to its ability to provide three-dimensional imaging, eliminating the superimposition of anatomical structures that can obscure lesions on two-dimensional radiographs. This enhanced spatial resolution allows for more accurate identification of subtle periapical changes, bone resorption, and complex root anatomy that may be missed on radiography. Similarly, in veterinary medicine, studies in dogs have suggested that performing intraoral radiography following CT is often unnecessary, as CT alone reliably captures clinically relevant dental pathology [29]. Therefore, in cases where detailed assessment of dental and periapical structures is critical—for instance, in animals with suspected complex or multifocal disease—CT should be considered the imaging modality of choice to ensure comprehensive evaluation and guide appropriate clinical management. While CT imaging is generally more expensive for the owner compared with conventional radiography, its superior ability to detect dental and periapical pathology can lead to more accurate diagnosis and targeted treatment. By identifying lesions that may be missed on X-rays, CT can help prevent misdiagnosis, reduce the need for repeat procedures, and minimize complications. In this way, the upfront cost of CT may ultimately result in lower overall expenses and improved outcomes for both the animal and the owner.

The weak agreement (57%) for overall suspicion of periapical infection in CT compared to radiography is lower than what was previously found in horses [22], where agreement among these two modalities was as high as 97%. Several factors likely contribute to this discrepancy. First, the unfamiliarity of interpreters with the radiographic anatomy of South American Camelids may have led to under-detection of abnormalities, as subtle periapical changes can be easily overlooked when clinicians are more accustomed to equine dental structures. Second, the relatively compact and densely packed cranial anatomy of alpacas results in significant superimposition of adjacent anatomical structures on radiographs, further limiting the visibility of individual tooth roots and periapical regions.

The authors acknowledge that the absence of histological confirmation represents a significant limitation of this study. Histology remains the definitive standard for diagnosing dental pathology, providing microscopic evidence of periapical lesions, inflammation, and other structural changes that imaging alone cannot fully resolve. Without histological validation, the ability to conclusively interpret imaging findings is inherently constrained, and subtle or early-stage pathological changes may be overlooked or mischaracterized. Nevertheless, given the partially retrospective nature of this study, it was not feasible to perform histopathology on all teeth. Despite this limitation, the study evaluates a substantial number of pathological teeth using advanced imaging modalities, generating important preliminary insights and guiding future work in which histological assessment can provide critical confirmation and enhance the precision of imaging-based diagnoses. Furthermore, the inclusion of control specimens could have given interesting insights into potential pathological findings in dental elements that were not clinically or radiographically targeted as pathological. Future studies that include histological samples and control cases would further strengthen the current findings.

## 5. Conclusions

In conclusion, the agreement between CT and radiography for the detection of the hallmark signs of periapical infection ranges from null to moderate, depending on the considered parameters, which is most likely the result of interpreting unfamiliar images and of the limitation imposed by superimposition when examining this region. Overall, CT identified more dental abnormalities than radiography, making it a valuable auxiliary diagnostic tool for the detection of periapical infections in cheek teeth in alpacas.

## Figures and Tables

**Table 1:** An overview of specimens’ sex, age, and number of dental elements included in the study.

Animal	Age	Sex	Number of Dental Elements Included in the Study
Alpaca 1	5 years 0 months	Neutered Male	8
Alpaca 2	1 year 1 month	Neutered Male	2
Alpaca 3	2 years 6 months	Female	5
Alpaca 4	4 years 2 months	Female	8
Alpaca 5	2 years 6 months	Female	4
Alpaca 6	9 years 9 months	Male	12
Alpaca 7	2 years 6 months	Neutered Male	8
Alpaca 8	3 years 0 months	Female	6
Alpaca 9	2 years 3 months	Female	4
Alpaca 10	9 years 2 months	Neutered Male	12
Alpaca 11	1 year 7 months	Male	4
Alpaca 12	8 years 0 months	Female	12
Alpaca 13	8 years 3 months	Neutered Male	12
Alpaca 14	10 years 4 months	Neutered Male	10
Alpaca 15	3 years 7 months	Neutered Male	8
Alpaca 16	8 years 0 months	Female	11
Alpaca 17	5 years 0 months	Female	12
Alpaca 18	11 years 5 months	Female	12
Alpaca 19	8 years 0 months	Neutered Male	8

**Table 2:** Inter-modality agreement.

Imaging Measure	Number of Tested Alpaca Teeth	Cohen’s Kappa [95% CI]	Percentage of Agreement Across Modalities	Strength of Agreement
Pulpar changes	158	0.026 [−0.032, 0.084]	3%	No agreement
Widened periodontal space	158	0.54 [0.4, 0.68]	54%	Weak agreement
Periapical halo	158	0.57 [0.36, 0.77]	57%	Weak agreement
Apical root clubbing	158	0.71 [0.55, 0.86]	71%	Moderate agreement
Cementoma/hypercementosis	158	0.14 [−0.05, 0.33]	14%	No agreement
Radicular changes	158	0.69 [0.55, 0.83]	69%	Moderate agreement
Periapical sclerosis	158	0.64 [0.52, 0.77]	64%	Moderate agreement
Detectable lamina dura	158	0.39 [0.24, 0.55]	39%	Weak agreement
Lysis/fragmentation of the roots	158	0.51 [0.31, 0.7]	51%	Weak agreement
Sequestration	158	0.34 [0.088, 0.58]	34%	Weak agreement
Cortical destruction	158	0.51 [0.28, 0.74]	51%	Weak agreement
Periosteal reaction	158	0.65 [0.48, 0.81]	65%	Moderate agreement
Mandibular expansion	158	0.63 [0.43, 0.82]	63%	Moderate agreement
Changes in the alveolar bone	158	0.68 [0.56, 0.8]	68%	Moderate agreement
Clinical crown fracture	158	0.56 [0.12, 1]	56%	Weak agreement
Suspicion of apical infection	158	0.57 [0.43, 0.71]	57%	Weak agreement

## Data Availability

The original contributions presented in the study are included in the article. Further inquiries can be directed to the corresponding author.

## Abbreviations

The following abbreviations are used in this manuscript:

CT	Computed tomography
ECVDI	European College of Veterinary Diagnostic Imaging
mAs	Milliamper seconds
kVp	Kilovoltage peak
